# Large Enriched Fragment Targeted Sequencing (LEFT-SEQ) Applied to Capture of *Wolbachia* Genomes

**DOI:** 10.1038/s41598-019-42454-w

**Published:** 2019-04-11

**Authors:** Emilie Lefoulon, Natalie Vaisman, Horacio M. Frydman, Luo Sun, Lise Voland, Jeremy M. Foster, Barton E. Slatko

**Affiliations:** 1 Molecular Parasitology Group, New England Biolabs, Inc, Ipswich, USA; 2 Department of Biology, Boston University, Boston, Massachusetts USA; 3 CAPES Foundation, Ministry of Education of Brazil, Brasília, DF 70040-020 Brazil; 4 National Emerging Infectious Diseases Laboratories, Boston University, Boston, Massachusetts, USA

**Keywords:** Genomic analysis, Molecular biology, Next-generation sequencing

## Abstract

Symbiosis is a major force of evolutionary change, influencing virtually all aspects of biology, from population ecology and evolution to genomics and molecular/biochemical mechanisms of development and reproduction. A remarkable example is *Wolbachia* endobacteria, present in some parasitic nematodes and many arthropod species. Acquisition of genomic data from diverse *Wolbachia* clades will aid in the elucidation of the different symbiotic mechanisms(s). However, challenges of *de novo* assembly of *Wolbachia* genomes include the presence in the sample of host DNA: nematode/vertebrate or insect. We designed biotinylated probes to capture large fragments of *Wolbachia* DNA for sequencing using PacBio technology (LEFT-SEQ: Large Enriched Fragment Targeted Sequencing). LEFT-SEQ was used to capture and sequence four *Wolbachia* genomes: the filarial nematode *Brugia malayi*, *w*Bm, (21-fold enrichment), *Drosophila mauritiana* flies (2 isolates), *w*Mau (11-fold enrichment), and *Aedes albopictus* mosquitoes, *w*AlbB (200-fold enrichment). LEFT-SEQ resulted in complete genomes for *w*Bm and for *w*Mau. For *w*Bm, 18 single-nucleotide polymorphisms (SNPs), relative to the *w*Bm reference, were identified and confirmed by PCR. A limit of LEFT-SEQ is illustrated by the *w*AlbB genome, characterized by a very high level of insertion sequences elements (ISs) and DNA repeats, for which only a 20-contig draft assembly was achieved.

## Introduction

A comprehensive understanding of symbiotic evolution remains challenging^1^. A remarkable example of the biological relevance and universality of symbiotic interactions is that of *Wolbachia* bacteria. The study of obligate intracellular alpha-proteobacteria *Wolbachia* symbiont and its host interactions provide a model system for analysis as they are present in a large fraction of invertebrate species on this planet, including nematodes, insects, mites, spiders and crustaceans^2–5^. While in arthropods they generally act as reproductive parasites^6^, in their filarial nematodes hosts they have generally taken an alternative evolutionary trajectory as strict mutualists, being obligate for adult and larval worm development and reproduction^7,8^. Underlying mechanisms of symbiosis remain largely elusive, although for some insects, a pair of genes (*cifA* and *cifB*) have been identified as part of the cytoplasmic incompatibility system, one of the arthropod reproductive manipulation phenotypes^9,10^. Comparative genomic analyses remains a viable strategy to identify candidate genes involved in *Wolbachia* symbioses^11^. Obtaining additional genomic data from a variety of *Wolbachia* clades will help elucidate the nature of the symbiotic mechanisms(s).

Currently, *de novo* sequence assembly of *Wolbachia* genomes often confronts several obstacles. First, it remains challenging to produce high quality genome sequences due to the presence of host DNA, which can complicate the assemblies because of low levels of *Wolbachia* sequence reads relative to host reads. Furthermore, the presence of lateral gene transfers (LGTs) from *Wolbachia* to the host genome can complicate assembly^12,13^ as observed for the *Wolbachia* genome of *Drosophila ananassae*^14^. One method to overcome the host DNA problem is to use targeted *Wolbachia* genome enrichment to capture large DNA fragments, recently developed for short-read paired-end technologies^15,16^.

A second challenge of *de novo* genome assembly is the presence of many long repetitive elements^17^ which inhibit correct assemblies. The first *Wolbachia* genome studies highlighted the presence of large amounts of repetitive DNA^18,19^. For example, at least 14% of the genome of *Wolbachia* from *D*. *melanogaster* (*w*Mel) is composed of repetitive DNA and insertion sequences^18^. Although present at different levels among strains^20^, *Wolbachia* genomes often contain numerous transposable elements (Insertion sequences (ISs) and group II introns) and prophage sequences^15,18^. As opposed to short-read paired-end technologies, long-read sequencing methods, such as PacBio or Nanopore, enable longer sequence reads, often through the repeats and thus can significantly improve *de novo* assembly^17^.

Here, we demonstrate a large fragment targeted enrichment capture method using SeqCap® EZ probes (Roche) and PacBio sequencing for *Wolbachia de novo* assembly (LEFT-SEQ - Large Enriched Fragment Targeted Sequencing). We tested this method on three different *Wolbachia* strain*s*: *w*Bm, from the nematode *Brugia malayi*, for which a previous complete genome sequence was available; *w*AlbB, from the mosquito *Aedes albopictus*, for which different draft genomes were available; and two isolates of *w*Mau, from *Drosophila mauritiana*, for which no genome draft was available.

## Results

### *De novo* assembly and coverage

The LEFT-SEQ method was implemented to capture relatively large DNA fragments for long-read NextGen sequencing to more efficiently enable genome assemblies. The library preparation workflow was optimized, in particular with an additional exonuclease treatment step, modified PCR conditions and lower ratio of AMPure® PB bead/DNA clean-up (Supplementary Methods 1 & Supplementary Fig. 1), and applied to insect or nematode samples harboring *Wolbachia* symbionts.

**Figure 1:**
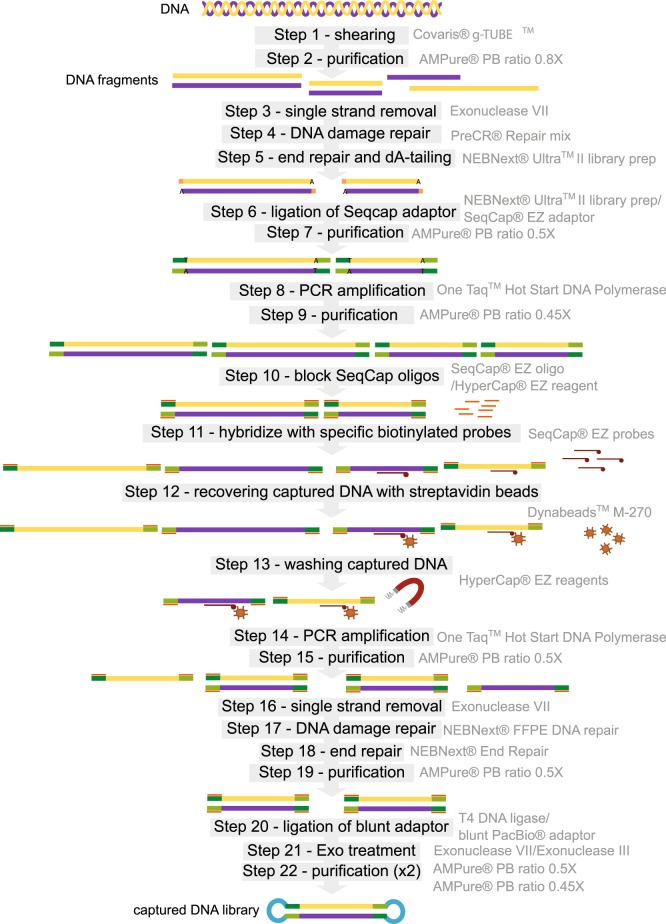
Workflow overview of LEFT-SEQ (Large Enriched Fragment Targeted Sequencing) library preparation.

**Figure 2:**
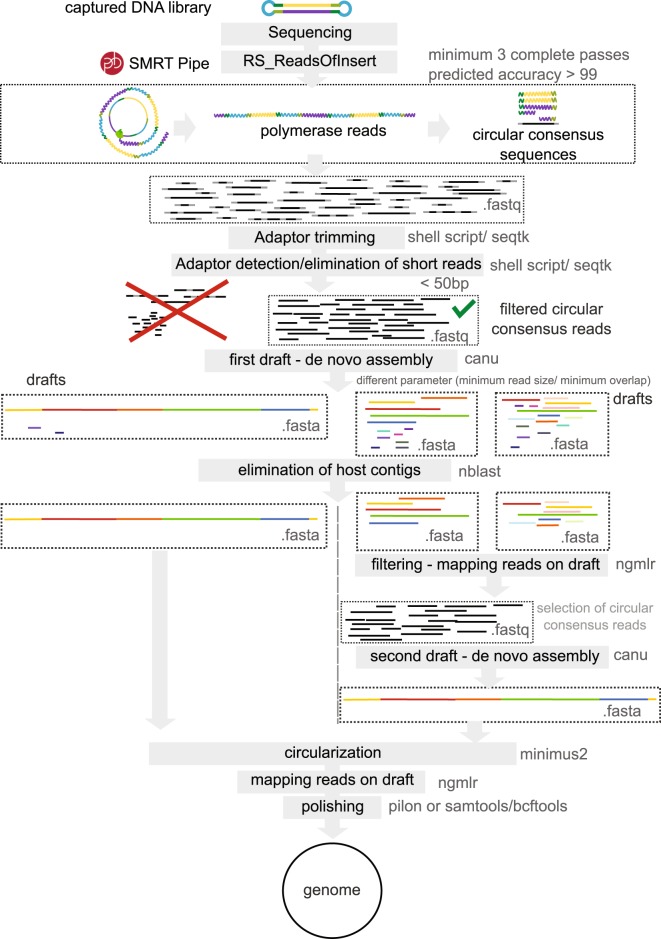
Overview of the bioinformatics pipeline.

We used LEFT-SEQ (Fig. 1) and bioinformatic analysis (Fig. 2) to produce *de novo* drafts of three *Wolbachia* symbiont genomes. The method created complete circular sequences for two of the genomes (*w*Bm, *w*Mau) and a set of 20 contigs for the third (*w*AlbB). For *w*Ma*u*, the *Wolbachia* from *D*. *mauritiana*, the analysis of 28,840 PacBio CCS (Circular consensus sequence) reads of fly population 177 and 45,984 CCS reads of the fly population 181 (3 SMRT cells each barcoded samples) produced, respectively, a circularized genome of 1,273,527 bp and 1,273,530 bp (Table 1). For *w*Bm, the *Wolbachia* from *B*. *malayi*, the analysis of 40,241 PacBio CCS reads (2 SMRT cells) produced a 3 contig draft while 76,216 reads (3 SMRT cells) produced a circularized genome of 1,080,939 bp (Table 1). For *w*AlbB, the *Wolbachia* from *Aedes albopictus*, the analysis of 81,233 reads (2 SMRT cells) produced a 42 contig draft and 290,028 PacBio CCS reads (12 SMRT cells) produced a 20 contig draft (Table 1). For *w*Bm and *w*Ma*u*, the single circular contigs were validated by PCR amplification (Tables S1, S2 and S3). Regarding genome coverage, enrichment provided a reduction in host sequences and only a few areas not covered at a depth of 20X (Table 1). The entire *w*Bm genome was captured at an average depth of 78X. Likewise, the entire *w*Mau genome was captured for both populations with average coverage of 71X for population 181 and 44X for population 177 (Table 1). The 20 contigs draft of *w*AlbB was obtained at an average depth of 266X (only 1.6% of the draft had coverage <20X) (Table 1).

**Table 1:** Information of produced genomes.

	*w*Bm		*w*Mau (pop 181)	*w*Mau (pop 177)	*w*AlbB	
Number of SMRT cell	2	3	3 (barcoded)	3 (barcoded)	2	12
Number of reads	40,241	76,216	45,984	28,840	81,233	290,028
Number of contigs	3	1	1	1	42	20
Size of the largest contig	416,288	1,080,939	1,273,588	1,273,527	186,312	249,386
Total length (bp)	1,082,170	1,080,939	1,273,588	1,273,527	1,466,139	1,492,731
N50	415,815	1,080,939	1,273,588	1,273,527	54,550	145,461
L50	2	1	1	1	7	4
Number of reads mapped to *wb* reference	23,742	45,107	NA	NA	61,930	246,276
% reads mapped to *wb* reference	59	59	NA	NA	76	84.9
Number of reads mapped to produced *wb*	23,733	45,103	44,734	27,842	71,203	249,032
Number of reads mapped to produced *wb*	59	59	97.28	96.5	87	85.8
Average depth	52X	78X	71X	44X	75X	266X
bases with coverage <20X	35,393	1,586	28,128	63,096	129,224	24,200
% bases with coverage <20X	3.2	0.14	2.2	4.9	8.5	1.6

Summary of *de novo* assembly using canu processed in the current study (statistics using QUAST^36^). Lines 1–7, summary statistics; lines 8 to 11, summary of mapping using ngmlr; lines 12 to 14, coverage statistics across the produced genomes using the SAMtools depth^38^. Abbreviations: bp: bases pair; *wb*: *Wolbachia;* pop: population.

### Efficiency of the enrichment

**Figure 3:**
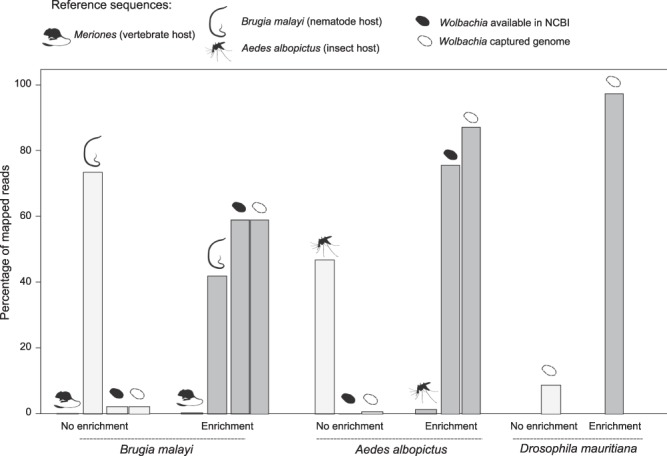
Evaluation of LEFT-SEQ enrichment method. The percentage of the reads mapped across different reference or draft genomes is reported for the three different samples without (pale grey) or with the enrichment method (dark grey). Host and *Wolbachia* symbionts are indicated with animal symbols.

The efficiency of the enrichment may impact the assembly quality if low levels of symbiont sequence reads are present, relative to host reads. The enrichment is more efficient for the *A*. *albopictus* sample than for the *D*. *mauritiana* or *B*. *malayi* samples (Fig. 3): 2.52% of the sequenced reads mapped to *w*Bm reference genome without enrichment versus 59% with the enrichment (23X increase); 8.96% of the sequenced reads mapped to produce the *w*Mau genome without enrichment vs. 97.28% with enrichment (11X increase); 0.2% of the sequenced reads mapped to *w*AlbB drafts or 0.95% to the produced draft without enrichment vs. an average 76.2% or 87.65% with the enrichment (90–340X increase) (Fig. 3). In terms of host-derived sequences, 73.99% of the reads mapped to host *B*. *malayi* reference without enrichment vs 41.98% with enrichment (1.76X decrease) with a few reads mapping to the jird (experimental mammalian host of the nematode) draft in both protocols. For *A*. *albopictus*, 47.24% of the reads mapped to the draft without enrichment compared to 1.56% with the enrichment (30X decrease). Thus, the variations among the *de novo* assemblies cannot be explained by different efficiencies of capture.

### Size of the sequenced reads

**Figure 4:**
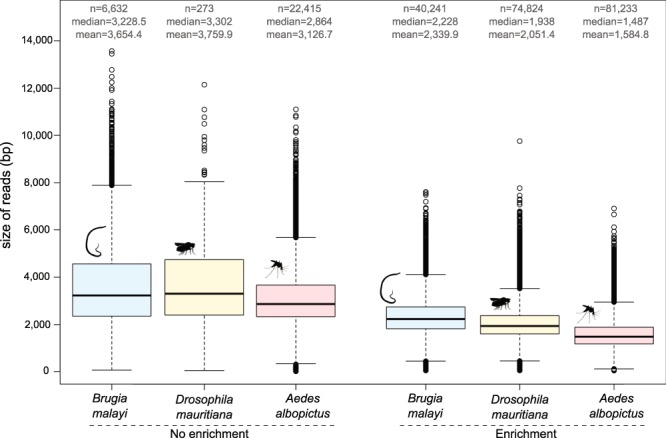
Box-and-whisker plot showing insert size for the three samples with the LEFT-SEQ method and the unenriched control. The different samples are indicated with color (blue for *B*. *malayi*, yellow for *D*. *mauritiana* and red for *A*. *albopictus*) and symbol. Small circles are outlier values. Additional statistics are indicated above the boxplot: number of analyzed reads, the median and the mean.

The current enrichment protocol was established after optimization described in the Supplementary files (Supplementary Methods 1) in order to maximize the size of sequenced reads. For all three genome samples, LEFT-SEQ reads are smaller (median between 1–1.3 kb shorter) than those of the control without the capture method (Fig. 4). Tests suggest that the observed shortening occurs during the hybridization bead capture step. Protocols without genomic DNA shearing and without the first PCR step show no clear difference in the median size of reads for all three tested genomes (Fig. S1). The current protocol includes the addition of an exonuclease VII treatment and a DNA damage repair step before the ligation, which reduces the formation of chimeric reads. SeqCap® adaptors (first ligation) are reduced to 3.6% and 6% with the optimized protocol from 14.7% of the reads for *B*. *malayi* and 11% for the *A*. *albopictus* sample after trimming (Fig. S1). Addition of one or two AMPure® PB bead clean-up steps before the annealing/binding to the SMRT templates increases the median size of the sequenced reads on the tested *A*. *albopictus* libraries, but the largest fragments appear to be lost during the library preparation (Fig. S1).

### Presence of mobile genetic elements

The RAST annotation of the *w*AlbB draft genome highlights the observation that each end of the 20 contigs encoded at least one mobile genetic element: either an insertion sequence element or a group II intron sequence. The number of insertion sequence elements (ISs) or transposases is highly variable among the studied *Wolbachia* genomes. In all, three partial ISs are detected in *w*Bm genome from two families, IS630 (n = 2) and IS1031 (n = 1); the open reading frames (ORF) lengths are respectively 103 bp and 62 bp (Table S1) and the same ISs are detected in the *w*Bm reference. 46 ISs are detected in *w*Mau from 17 different IS families, with the most represented being IS110 (47.83% of total detected ISs, maximum 1,122 bp), IS5 and IS6 (17.39%, minimum 186 bp) (Table S1). 209 ISs are detected in the *w*AlbB draft (Table S1), (ORFs from 180 bp to 1,320 bp). The most represented IS families are IS982 (46.86%), IS481 (36.36%), IS66 (8.61%) and IS3 (5.26%). Surprisingly, when the same analysis is performed on three previously published *w*AlbB drafts, variations are observed: 6 ISs for the 156 contig draft *wAlbB* (ASM24241v3), 9 ISs for the 131 contig draft *w*AlbB (ASM237914v1) and 7 ISs for the 177 contig draft *w*AlbB (ASM237484v1). The difference is more striking for group II intron genes: none are detected in *w*Bm, as previously observed^19,20^, 7–10 genes are detected in *w*Mau while 72 genes are detected in the *w*AlbB contigs. Thus, this high level of mobile genetic elements in *w*AlbB genome compared to the *w*Mau or *w*Bm genomes could explain the inability to generate a single complete consensus sequence.

### Detection of Single-nucleotide polymorphisms

There are several possible sources of error associated with NextGen sequencing protocols, including PCR errors, inherent DNA sequencing errors due to the chemistry^21,22^ or errors due to the *de novo* assembly process^23^. In order to test the accuracy of assembly based on PacBio CCS reads, we compared *w*Bm assemblies processed with different correction methods (described in Methods) with the reference *w*Bm genome available in the database. Variant detection between the *w*Bm assemblies of the current study and the reference *w*Bm genome indicates 8 transitions, 9 transversions and 1 insertion, independent of the application of polishing steps. These differences were confirmed by PCR amplification and sequencing (Fig. S2; Tables S2, S3 and S5). Using the two correction methods, only the deletion detection was variable between them. 21 deletions are in common among the *w*Bm assemblies relative to the *w*Bm reference. It is interesting to note that all the deletions occurred at regions where the identical nucleotide was repeated. Correction using SAMtools/BCFtools provided an assembly most similar to the reference, with fewer deletion events than the correction using Pilon, which had indicated more deletions (54, including 10 representing a total of 533 bp). These polishing methods were developed for use with Illumina data and not PacBio consensus sequences. As the coverage with the Pacbio CCS (circular consensus sequence) is low (78X coverage for *wBm*), it might explain why better results were obtained with the tuned SAMtools pipeline, known to reduce the effect of reads with excessive mismatches (http://samtools.sourceforge.net/mpileup.shtml). Comparison of the two assemblies of *w*Mau polished with the tuned SAMtools pipeline identified 19 base differences (Table S4), mainly deletion events, which all occurred at repeated bases and only one base mutation event. Four of these differences were tested by PCR amplification and all indicated absence of mutation (Table S5), suggesting these were sequencing read consensus errors.

## Discussion

LEFT-SEQ provides an efficient enrichment method for long read sequences derived from the endosymbiont *Wolbachia*. This enables the production of complete or almost complete *Wolbachia* genomes amongst a background of host sequences in a stepwise and efficient process. The efficacy of the method is variable according to the *Wolbachia* strain, as complete genomes of *w*Bm or *w*Mau were produced while only a 20-contig draft *w*AlbB genome was obtained, due to the presence of a high percentage of repeats in the genome. The analysis of *w*Bm shows that the method can enable SNP detection between samples, as at least 18 SNPs were confirmed by PCR, relative to the original published sequence. However, it is difficult to establish if they are real *de novo* differences or sequence errors during assembly of the original sequenced genome^19^. The original *w*Bm genome was completed by Sanger dideoxy sequencing of subclones derived from overlapping bacterial artificial chromosome (BAC) templates^19^ and it is conceivable this cloning approach introduced a small number of errors. However, while the *w*Bm samples derived from the same source, there is an approximate 20-year gap difference in time between isolation of the DNA samples for sequencing. Only 1 potential missense mutation was observed between the two sequenced *w*Mau genome samples but PCR amplification indicated that this was a sequencing error. These two lineages derived from the same initial mating 8 years before samples were collected for this study, suggesting there was no sequence diversity during this relatively short time frame. It is interesting to point out that PacBio reads are not commonly used for SNP detection or are at least rarely used without polishing with Illumina reads, due to the differences of error rates (often established as <0.8% for Illumina and around 10% for PacBio single-molecule reads)^22,24^. However as recently reported^25,26^, the use of PacBio circular consensus reads increases accuracy, as used in the current study.

The limitation of the method to assemble the *w*AlbB genome is not related to the enrichment efficiency or coverage depth. Indeed, a higher percentage of reads belonging to *Wolbachia* was observed for the mosquito sample, compared to the nematode sample (Fig. 3). This efficiency difference among the samples may be due to *Wolbachia* copy number differences in their hosts or a differential number of LGTs, but in each case, LEFT-SEQ provides a highly specific sequence capture. The presence of repetitive elements is very variable among analyzed *Wolbachia*^15,18,19^. For cases with a very high percentage of repeats, an increased number of reads even with somewhat longer read lengths improved *de novo* assembly, but still did not enable the assemblers to produce a complete genome. Even longer fragments will be required to cross the repeats in situations like this. Along these lines, LEFT-SEQ identified a high number of mobile elements (ISs) and group II intron-associated genes in our *w*AlbB draft, as compared to the previous submissions.

The different protocols tested during the study to attempt to obtain larger fragments for PacBio sequencing suggested that the fragment length obtained during the library preparation (average 2.3 kb for *B*. *malayi*; 2 kb for *D*. *mauritiana* and 1.6 kb for *A*. *albopictus*) is not related to the initial fragmentation or PCR amplification steps (Fig. S1). We suspect the limiting step may be in the bead hybridization step where longer DNA fragments are selectively eliminated either due to multiple probes hybridizing on the same large DNA fragment or shearing due to large DNA interacting with the beads. Even when size selection systems are utilized, (*e*.*g* Sage ELF, Sage Science, Beverly MA) size selection) which can increase the average read size^27^, the problem still remains and while more DNA input might be helpful to increase the average DNA size for capture, this may be problematic for many studies, where DNA is limited. A future goal will be the modification of the capture step for longer fragment isolation (enhanced, LEFT-E-SEQ).

## Materials and Methods

### Source of materials

Three invertebrate species were used to test the method of *Wolbachia* long DNA fragment capture: the filarial nematode *B*. *malayi* grown in *Meriones unguiculatus* (Mongolian jird is the experimental mammalian host) naturally infected with *w*Bm (TRS Labs, Georgia, USA), the fruit fly *Drosophila mauritiana* infected with *w*Mau (flies from Frydman Lab, Boston University, lab stocks 177 and 181 generated by single pair crosses from the same *w*Mau infected stock)^28^ and the mosquito *Aedes albopictus* with an artificial single-infection by *w*AlbB (mosquitoes from the Rasgon lab, Pennsylvania State University)^29^. The DNA of the different samples was extracted using the DNeasy Blood and Tissue kit following the manufacturer’s recommendations (Qiagen, Germany) with overnight incubation at 56 °C with proteinase K. DNA was eluted into 1X TE (10 mM Tris-HCl, 1 mM EDTA, pH 8.0). The number of specimens pooled for the DNA extraction was variable: 12 female nematodes for *B*. *malayi*, 10 female flies for *D*. *mauritiana* and 5 mosquitoes for *A*. *albopictus*. In the case of *D*. *mauritiana*, two different lineages were sequenced using the same hybridization reaction (see below). Each of these two lineages came from a single pair mating derived from the same *w*Mau infected stock in 2010.

### Design of capture-based target enrichment DNA probes

The targeted DNA probes were designed by Roche NimbleGen (Madison, US) based on 25 complete or draft *Wolbachia* sequences (total of 121,300 Tiled regions: average size 997 bp): *Wolbachia* endosymbiont of *Pratylenchus penetrans wPpe* (ASM175266v1;GCF_001752665.1); *Wolbachia* endosymbiont of *B*. *malayi* (ASM838v1;GCF_000008385.1); *Wolbachia* endosymbiont of *Onchocerca ochengi wOo* (ASM30688v1;GCF_000306885.1); *Wolbachia* endosymbiont of *O volvulus* (W_O_volvulus_Cameroon_v3;GCF_000530755.1); *Wolbachia* endosymbiont of *Dirofilaria immitis* (wDiv2; http://dirofilaria.nematod.es); *Wolbachia* endosymbiont of *Litomosoides sigmodontis* (wLs2; http://litomosoides.nematod.es); *Wolbachia* endosymbiont of *Wuchereria bancrofti* (ASM33839v1;GCF_000338395.1); *Wolbachia* endosymbiont of *Cimex lectularius wCle* (ASM82931v1;GCF_000829315.1); *Wolbachia* endosymbiont of *Culex quinquefasciatus Pel wPip* (ASM7300v1;GCF_000073005.1); *Wolbachia* endosymbiont of *D*. *melanogaster wMel* (ASM802v1;GCF_000008025.1); *Wolbachia* endosymbiont of *D*. *simulans wRi/wNo*/*wHa/wAu* (ASM2228v1;GCF_000022285.1/ ASM37658v1;GCF_000376585.1/ASM37660v1;GCF_000376605.1/Wau001;GCF_000953315.1); *Wolbachia* endosymbiont of *D*. *simulans* (ASM16749v1;GCA_000167495.1); *Wolbachia* endosymbiont of *Diaphorina citri* (wACP3;GCF_000331595.1); *Wolbachia* endosymbiont of *D*. *ananassae* (ASM16747v1;GCF_000167475.1); *Wolbachia* endosymbiont of *D*. *willistoni* (ASM15358v1;GCF_000153585.1); *Wolbachia* endosymbiont of *D*. *suzukii* (ASM33379v2;GCF_000333795.1); *Wolbachia* endosymbiont of *Muscidifurax uniraptor wUni* (ASM198363v1;GCF_001983635.1); *Wolbachia* endosymbiont of *Hypolimnas bolina wBol1-b* (ASM33377v1;GCF_000333775.1); *Wolbachia* endosymbiont of *Glossina morsitans* (wGmm_version4;GCF_000689175.1); *Wolbachia* endosymbiont of *Nasonia vitripennis wVitA/wVitB* (ASM198361v1;GCF_001983615.1/WVB_1.0;GCF_000204545.1); *Wolbachia* endosymbiont of *A*. *albopictus w*AlbB (ASM24241v3;GCF_000242415.2).

### Library preparation protocol

#### DNA fragmentation

DNA was fragmented using a Covaris® g-TUBE^TM^ (Covaris, US) to produce 8-kb fragments. About 1μg DNA (quantified by Nanodrop) was centrifuged twice at 6,500 rpm (Fig. 1). The sheared DNA was purified using 0.8X AMPure® PB beads (PacBio, US) to remove smaller fragments. Elution was in a 57 μL volume of 1X TE.

### DNA repair and large insert library preparation

Large insert libraries were constructed using an adaptation of NEBNext® Ultra^TM^ II DNA Library Prep protocol (New England Biolabs, US) with preliminary steps of single strand DNA elimination using Exonuclease VII treatment and DNA damage repair using PreCR® repair mix (New England Biolabs, US) (Fig. 1). A 55 μL reaction volume contained the fragmented DNA (~500 ng), 6 μL of NEBNext® Ultra^TM^ II End Prep Reaction buffer, 1 μL of NAD+, and 1 μL of Exonuclease VII and was incubated 15 minutes at 37 °C. 2 μL of PreCR® enzyme mix was added to the reaction and incubated for 30 minutes at 37 °C. The sheared DNA was end repaired and A-tailed by addition of 3 μL of NEBNext® Ultra^TM^ II End Prep Enzyme Mix and incubated for 5 minutes at 25 °C and then 30 minutes at 65 °C. Following this, SeqCap® adaptors (Roche, NimbleGen) were ligated to both ends of DNA using the NEBNext® Ultra^TM^ II Ligation Module (New England Biolabs) (Fig. 1). A 96 μL reaction volume contained 60 μL of the end repaired reaction mixture (previous step), 30 μL NEBNext® Ultra^TM^ II Ligation Master Mix, 1μL NEBNext® Ligation Enhancer and 4 μL SeqCap® Adapter A (10 μM stock). The reaction was incubated 20 °C for 15 minutes followed by a 0.5X AMPure® PB bead clean-up and elution in 27 μL water. 1μL amplified DNA was electrophoresed using a DNA 12,000 chip on the 2100 Bioanalyser system (Agilent, US) to determine the concentration.

### Library amplification

The resultant insert library was PCR amplified using One Taq^TM^ Hot Start DNA Polymerase (New England Biolabs) (Fig. 1) in a 25 μL reaction containing: Adaptor Ligated DNA Fragments (between 50 to 150 ng), 0.5 μM of each PCR oligo (PCR oligo 1-5′-AAT GAT ACG GCG ACC ACC GAG A- and PCR oligo 2-5′-CAA GCA GAA GAC GGC ATA CGA G-), 1X OneTaq Buffer, Mg-free, 1.5 mM MgCl_2_, 0.4 mM each dNTP, 2.5 U OneTaq Hot Start enzyme. PCR was performed using the following conditions: 94 °C for 2 minutes, 7 cycles of 94 °C 20 seconds, 56 °C 20 seconds and 68 °C 8 minutes, followed by 68 °C for 10 minutes. The amplified DNA was purified by a 0.45X AMPurePB bead (PacBio) purification. 1 μL amplified DNA was electrophoresed using a DNA 12,000 chip with the 2100 Bioanalyser system (Agilent, US) to determine the concentration.

### Target enrichment hybridization

1μg of the library, 10 μL SeqCap EZ Developer Reagent (Roche NimbleGen,), 1 μL SeqCap HE Universal Oligo (1 mM) and 1μL SeqCap HE Index Oligo (1 mM) were combined and vacuum dried at 60 °C. The hybridization of DNA with EZ library probes was performed according to SeqCap EZ HyperCap protocol (NimbleGen, User’s guide v1.0) (Fig. 1). However, Dynabeads^TM^ M-270 Streptavidin beads (Invitrogen, US) were used to capture the DNA. The captured DNA fragments were then amplified with the same PCR conditions as the first PCR with the only difference being that the number of cycles was increased to 15. The amplified DNA was purified with 0.5X AMPurePB beads. 1 μL amplified DNA was electrophoresed using a DNA 12,000 chip with the 2100 Bioanalyser system (Agilent) to analyze capture success.

### PacBio library preparation

A preliminary step of single strand removal and DNA damage repair was performed using Exonuclease VII and the NEBNext® FFPE DNA Repair kit (New England Biolabs, MA, US) in a 48 μL reaction volume containing: the captured DNA (~500 ng), 5 μL of NEBNext® FFPE DNA repair buffer and 1 μL of exonuclease VII (NEB) (Fig. 1). The reaction was incubated 15 minutes at 37 °C. 2 μL of NEBNext® FFPE DNA Enzyme mix was added to the reaction and incubated 20 minutes at 37 °C. 5 μL of NEBNext® End Repair enzyme mix was then added to the reaction and incubated at 25 °C for 5 minutes. This was followed by a 0.45X AMPurePB bead clean-up step. Next, PacBio Blunt Adapters were ligated in a 40 μL reaction volume containing the end repaired reaction mixture of the previous step using 0.5 μM Annealed PacBio Blunt Adapters, 1X NEB T4 Ligase buffer and 2,000 units of T4 DNA Ligase. The reaction was incubated at 25 °C for 1 hour and at 65 °C for 10 minutes to inactivate the ligase. 100 units of Exonuclease III (NEB) and 10 units of Exonuclease VII NEB) were added to the reaction and incubated 37 °C for 45 minutes. This was followed by one 0.5X AMPure® PB bead clean-up and a second 0.45X AMPure® PB bead clean-up. The size and the concentration of the library was assayed on an Agilent Bioanalyzer using a DNA 12,000 chip according to manufacturer’s instructions.

Annealing and binding to the produced PacBio SMRTbell Template was performed according to the manufacturer’s recommendations (PacBio, US). For each sample, a control library without capture was also produced: only the shearing and the PacBio library preparation steps were utilized.

### Bioinformatics analysis

PacBio circular consensus sequences (CCS) were generated using SMRT® pipe RS_ReadsOfInsert Protocol (PacBio) with a minimum 3 full passes and minimum predicted accuracy superior at 99 (Fig. 2). It was first necessary to remove the SeqCap adapter sequences by trimming off the first and last 65 bp of the reads using seqtk (github.com/lh3/seqtk) (Fig. 2). Reads smaller than 20 bp and reads containing residual adaptor sequences (potential chimeric reads) were detected and removed using seqkt (analyses were performed with an in-house shell script). The size of reads was calculated and their means, medians and boxplots were analyzed using R^30^.

A first *de novo* assembly was done using Canu^31^ with the standard overlap algorithm by varying the minimum reads length (100 to 2,200 bp) and the minimum overlap length (100 to 2,000 bp) (Fig. 2). The contigs belonging to *Wolbachia* symbionts were identified by nucleotide similarity using BLASTn^32^. If multiple contigs were obtained, a filtering was performed: the circular consensus sequences (CCS) were mapped against the best produced draft (having the largest contig size and/or the highest total length) using ngmlr^33^ with the PacBio preset settings (Fig. 2). A second *de novo* assembly was performed with the new selection of CCS using Canu^31^. If a single large contig was produced after the first original assembly, this selection step was not performed.

Successful final assemblies should produce a single large contig with the beginning and end of the genome assembly containing a duplicate sequence. To create a circularized genome, a “break” was introduced in the single contig and minimus2 (modified version of the minimus pipeline^34^) was used to detect overlaps and join the ends of the two contigs (Fig. 2). The final step was error correction of the draft. The CCS reads were once again mapped against the produced circularized genome using ngmlr^33^. Tests of polishing were performed to optimize the consensus sequence calling. Two methods were used: one using pilon^35^ in order to identify misassemblies and detect variants and a second using SAMtools and BCFtools (the parameter was tuned to reduce the effect of reads with excessive mismatches) (http://samtools.sourceforge.net/mpileup.shtml) (Fig. 2). Assembly statistics were evaluated using QUAST^36^. To analyze the polishing, the produced drafts and the genome reference were aligned using progressiveMauve^37^. PCR primers were designed to confirm the sites of circularization of the single contigs, as well as any sequences containing potential polymorphisms observed between the produced genome sequence and database references, when available (Table S5).

Evaluation of the enrichment was established by mapping each CCS against genome references and produced genomes using ngmlr^33^ with the PacBio preset settings (Fig. 2). In the case of *B*. *malayi*, the available *w*Bm complete genome ASM838v1 (NC_006833)^19^ was used. In the case of *A*. *albopictus*, mapping was tested with the available different drafts: *w*AlbB ASM24241v3 (156 contigs, N50 = 12,719; 1,162,431 bp), ASM237914v1 (131 contigs, N50 = 12,474; 1,176,060 bp) and ASM237484v1 (177 contigs, N50 = 11,063; 1,517,743 bp) (direct NCBI submission). Unlike the two other samples, the enrichment for *D*. *mauritiana* was established mapping with the assembly of the current study because no reference was available. The coverage of the assembly was evaluated with SAMtools (samtools depth)^38^. In order to study the limitations of the assemblies, the processed genomes sequence or drafts were analyzed using RAST^39^. Transposase elements were identified: insertion sequences (ISs) using ISSAGA^40^ and group II introns were annotated by the RAST algorithm.

## Supplementary information

Supplementary information

## Data Availability

Data generated are available in GenBank: BioProject PRJNA508212; BioSample SAMN10519683 for *Wolbachia* endosymbiont strain TRS of *Brugia malayi* (genome: CP034333); BioSample SAMN10519763 and SAMN10519765 for *Wolbachia* endosymbiont of *Drosophila mauritiana* strain (respectively *w*Mau lineages 177 and 181) (genome: CP034334 and CP034335); BioSample SAMN10519629 for *Wolbachia* endosymbiont of *Aedes albopictus w*AlbB (genome: RWIK00000000). The raw data are available in GenBank as Sequence Read Archive (SRA): SRR8283319 to SRR8283325.
