# Author Correction: Large Enriched Fragment Targeted Sequencing (LEFT-SEQ) Applied to Capture of *Wolbachia* Genomes

**DOI:** 10.1038/s41598-019-55305-5

**Published:** 2019-12-24

**Authors:** Emilie Lefoulon, Natalie Vaisman, Horacio M. Frydman, Luo Sun, Lise Voland, Jeremy M. Foster, Barton E. Slatko

**Affiliations:** 1 Molecular Parasitology Group, New England Biolabs, Inc, Ipswich, USA; 2 Department of Biology, Boston University, Boston, Massachusetts, USA; 3 CAPES Foundation, Ministry of Education of Brazil, Brasília, DF 70040-020 Brazil; 4 National Emerging Infectious Diseases Laboratories, Boston University, Boston, Massachusetts, USA

Correction to: *Scientific Reports* 10.1038/s41598-019-42454-w, published online 11 April 2019

Lise Voland was omitted from the author list in the original version of this Article. This has been corrected in the PDF and HTML versions of the Article, and in the accompanying Supplementary Information file.

The Author Contributions section now reads:

E.L., L.S. and B.E.S. conceived and designed the experiments. E.L. L.V. and N.V. performed the experiments. E.L. analyzed the data. H.F. contributed materials. E.L., J.M.F. and B.E.S. wrote the main manuscript text. All authors reviewed the manuscript.

The Competing Interests section now reads:

E.L., L.S., J.F., L.V. and B.S. are employed by New England Biolabs, Inc., who provided funding for this project.

