# Supplementary material for: Large Enriched Fragment Targeted Sequencing (LEFT-SEQ) Applied to Capture of Wolbachia Genomes
Source: Sci Rep. 2019 Apr 11;9:5939. doi: 10.1038/s41598-019-42454-w (PMC6459864; doi:10.1038/s41598-019-42454-w)
Supplement: Supplementary file 1 — Supplementary information [file 41598_2019_42454_MOESM1_ESM.docx]

**Large Enriched Fragment Targeted Sequencing (LEFT-SEQ) Applied to Capture of *Wolbachia* Genomes**

Emilie Lefoulon* (1), Natalie Vaisman (2,3), Horacio M. Frydman (2,4), Luo Sun (1), Lise Voland (1), Jeremy M. Foster (1) &, Barton E. Slatko (1)

(1) Molecular Parasitology Group, New England Biolabs, Inc., Ipswich, United States of America;

(2) Department of Biology, Boston University, Boston, Massachusetts, United States of America;

(3) CAPES Foundation, Ministry of Education of Brazil, Brasília - DF 70040-020, Brazil

(4) National Emerging Infectious Diseases Laboratories, Boston University, Boston, Massachusetts, USA

*corresponding author:

Emilie Lefoulon

e-mail: elefoulon@neb.com

Phone: +1 978 998 8975; Fax: +1 978-921-1350

**Supplementary information**

**Supplementary Methods 1:** Protocol optimization

Variations of this basic protocol were tested to increase the average size of templates for the sequencing reactions. A protocol without the Exonuclease VII treatment and DNA Damage repair steps (before the end repair steps; see step 3 and 4 in Figure 1) and with AMPure® PB bead clean-up at a higher bead ratio (0.8X) (for all the steps excluding the two last purifications using 0.5X ratio; step 2, 7, 9, 15, 19 and 22 in Figure 1) was tested with *B. malayi* and *A. albopictus* samples. A protocol with a supplementary 0.45X AMPure® PB bead clean-up before the Annealing and Binding of the SMRTbell Template was also performed with the *A. albopictus* sample (after step 23 in Figure 1). A protocol without fragmentation of genomic DNA and without the first PCR was tested with all three samples (step 1,2, 7 and 8 in Figure 1).

**Supplementary Figure 1:** Boxplot with whiskers of insert size for the three samples with different LEFT-SEQ protocols.


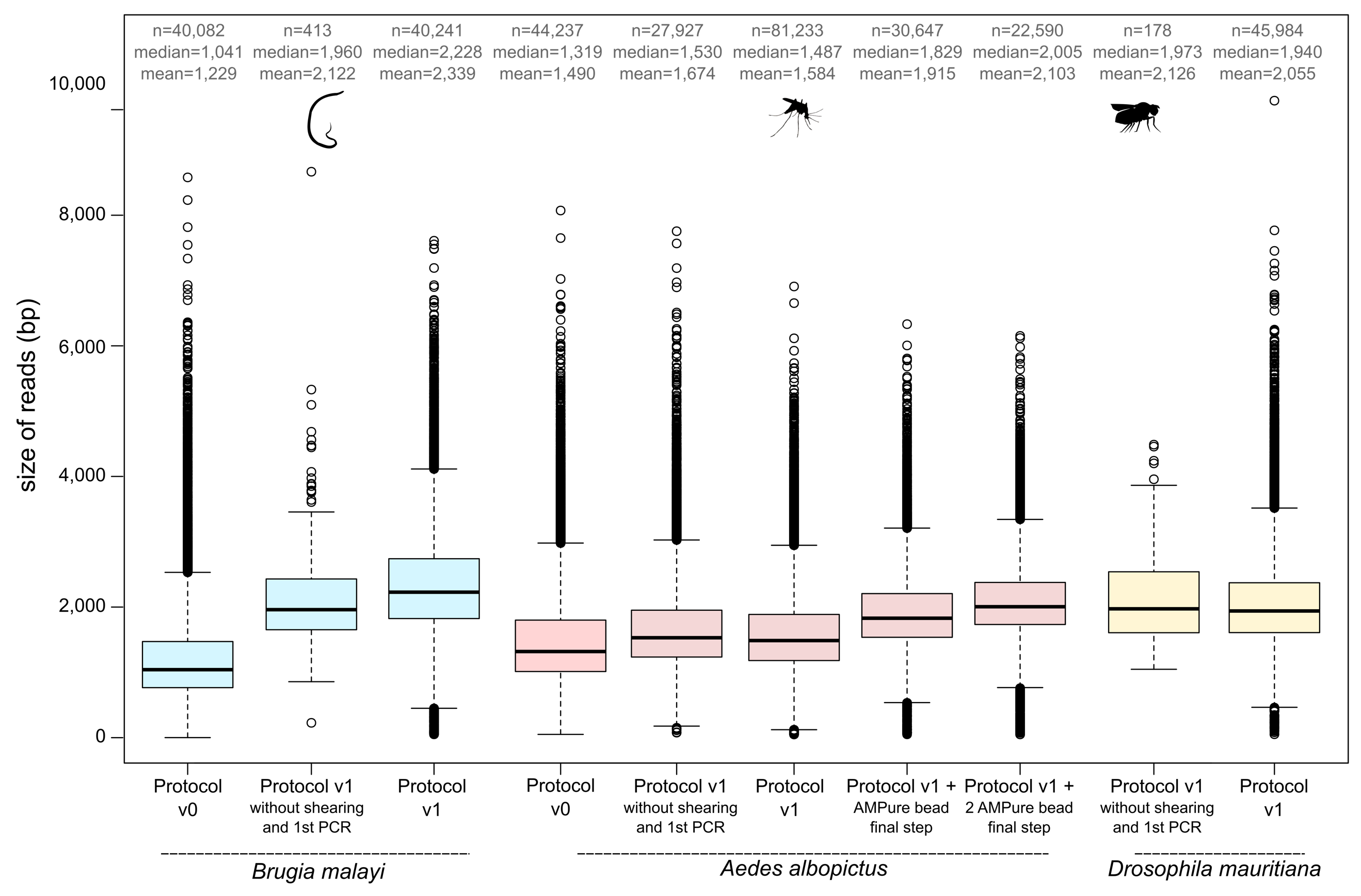


The different samples are indicated with color (blue for *Brugia malayi*, yellow for *Drosophila mauritiana* and red for *Aedes albopictus*). Result of reads produced with the different protocols: protocol v0 is the same but without the Exonuclease VII treatment and DNA Damage repair steps (before the end repair steps) and with AMPure bead clean-up at a higher bead ratio (0.8X) (for all the steps excluding the two last purification using 0.5X ratio). Protocol v1 is the one described in the current study. Modifications of this protocol (v1) without the shearing step and the first PCR or with a supplementary 0.45X AMPure PB beads clean-up before the Annealing and Binding of the SMRTbell Template are also presented, performed with the *Aedes albopictus* sample. The additional statistics are indicated above the boxplot: number of analyzed reads, the median and the mean.

**Supplementary Figure 2:** Observed differences between the *wBm* reference (ASM838v1; GCF_000008385.1) and the assemblies of the current study, without and with correction.


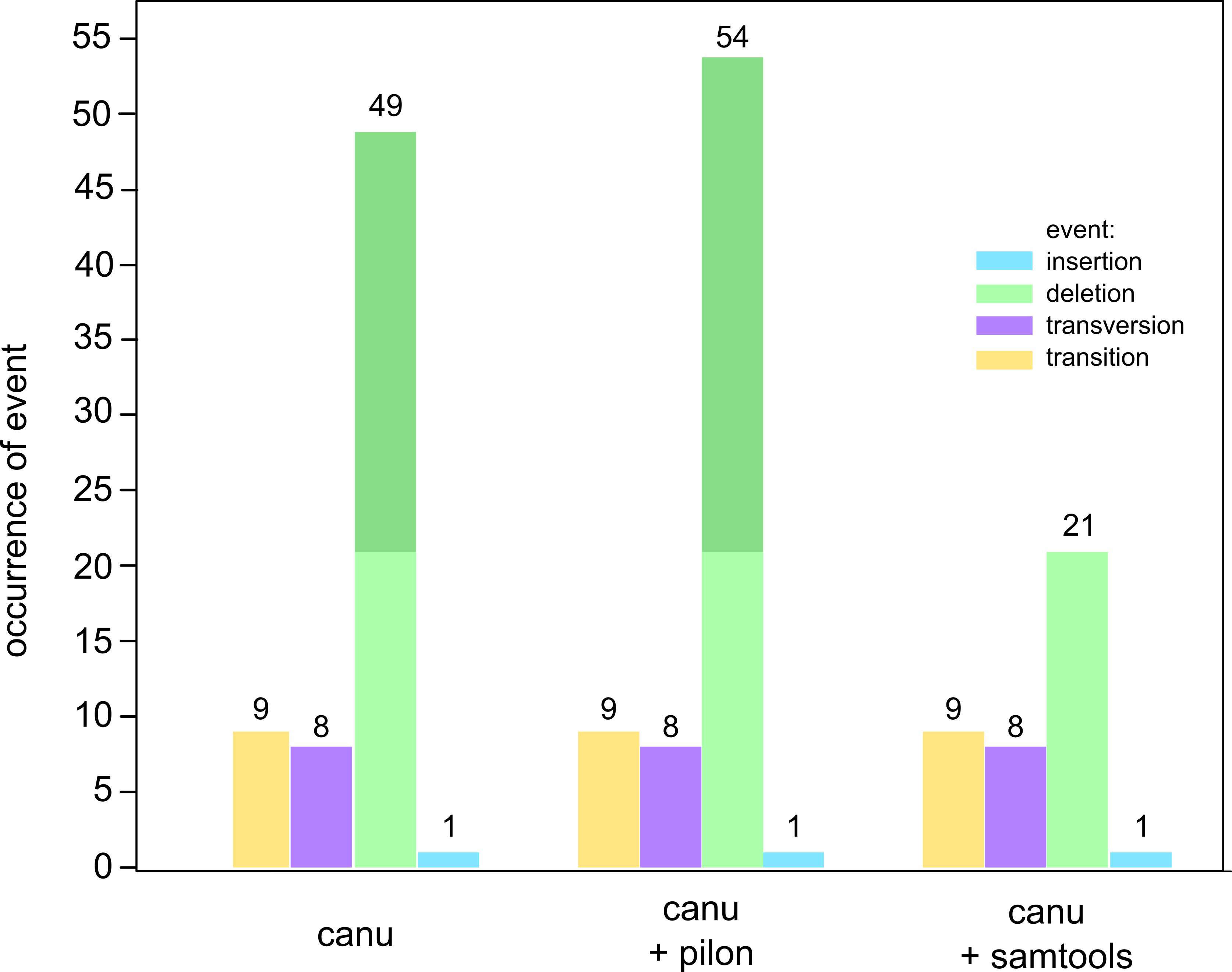


The same data has been used for assemblies processing but the bioinformatics analysis is different. These different bioinformatics analyses are indicated by the X axis: assembly labeled “canu” is the assembly using canu software without correction; “canu + pilon” is the assembly using canu and then polished using pilon software; “canu + samtools” is the assembly using canu and then polished using the tuned SAMtools pipeline (see Methods). The light color indicates difference from the reference that are common to all assemblies.

**Supplementary Table S1:**

Summary of insertion sequence (IS) detection using ISSAGA. The first column indicates the analyzed *Wolbachia* strain. The following columns indicate the name of the ORF, the percentage of similarity, the family of IS, the start of the ORF (orf_L), the end of the ORF (orf_R), the length of the ORF in base pairs, the length of the ORF in amino acids (aas) and orientation of the strand (ori).

| **Wolbachia strain** | **similarity** | **family** | **orf_L** | **orf_R** | **orf_bp** | **aas** | **ori** |
| --- | --- | --- | --- | --- | --- | --- | --- |
| *wBm* | 77.22% ISWpi10_aa2 | IS630 | 722114 | 722425 | 312 | 103 | + |
| *wBm* | 85.48% ISWpi1_aa2 | IS5 ssgr IS1031 | 723898 | 724086 | 189 | 62 | + |
| *wBm* | 77.22% ISWpi10_aa2 | IS630 | 814510 | 814821 | 312 | 103 | + |
| *wMau* | 58.35% ISGme8_aa1 | IS110 | 39635 | 40600 | 966 | 321 | + |
| *wMau* | 93.15% ISWpi14_aa3 | IS110 | 59392 | 60489 | 1098 | 365 | + |
| *wMau* | 93.15% ISWpi14_aa3 | IS110 | 110836 | 111855 | 1020 | 339 | + |
| *wMau* | 73.17% ISAcma25_aa2 | IS630 | 171440 | 171691 | 252 | 83 | + |
| *wMau* | 91.54% ISCaa6_aa1 | IS5 ssgr ISL2 | 195530 | 195276 | 255 | 84 | - |
| *wMau* | 84.05% ISCaa6_aa1 | IS5 ssgr ISL2 | 196101 | 195478 | 624 | 207 | - |
| *wMau* | 93.15% ISWpi14_aa3 | IS110 | 198625 | 197606 | 1020 | 339 | - |
| *wMau* | 76.11% ISCca2_aa1 | IS6 | 212050 | 211865 | 186 | 61 | - |
| *wMau* | 91.59% ISWpi12_aa1 | IS110 | 225320 | 225691 | 372 | 123 | + |
| *wMau* | 91.91% ISWpi12_aa1 | IS110 | 225645 | 226289 | 645 | 214 | + |
| *wMau* | 76.11% ISCca2_aa1 | IS6 | 233323 | 233508 | 186 | 61 | + |
| *wMau* | 84.11% ISCaa6_aa1 | IS5 ssgr ISL2 | 245936 | 246769 | 834 | 277 | + |
| *wMau* | 93.15% ISWpi14_aa3 | IS110 | 250382 | 251401 | 1020 | 339 | + |
| *wMau* | 74.62% ISCca2_aa1 | IS6 | 254864 | 255049 | 186 | 61 | + |
| *wMau* | 78.08% ISCca8_aa1 | IS6 | 266448 | 266218 | 231 | 76 | - |
| *wMau* | 76.11% ISCca2_aa1 | IS6 | 266875 | 266690 | 186 | 61 | - |
| *wMau* | 69.33% IS1230B_aa2 | IS3 ssgr IS3 | 349688 | 349437 | 252 | 83 | - |
| *wMau* | 72.58% ISThsp4_aa2 | IS3 ssgr IS3 | 349896 | 349702 | 195 | 64 | - |
| *wMau* | 66.27% ISNmu1_aa1 | IS3 ssgr IS3 | 350451 | 350191 | 261 | 86 | - |
| *wMau* | 58.35% ISGme8_aa1 | IS110 | 362583 | 361618 | 966 | 321 | - |
| *wMau* | 76.11% ISCca2_aa1 | IS6 | 379661 | 379846 | 186 | 61 | + |
| *wMau* | 78.08% ISCca8_aa1 | IS6 | 380088 | 380318 | 231 | 76 | + |
| *wMau* | 76.11% ISCca2_aa1 | IS6 | 383499 | 383684 | 186 | 61 | + |
| *wMau* | 78.08% ISCca8_aa1 | IS6 | 383926 | 384156 | 231 | 76 | + |
| *wMau* | 76.11% ISCca2_aa1 | IS6 | 386473 | 386658 | 186 | 61 | + |
| *wMau* | 78.08% ISCca8_aa1 | IS6 | 386900 | 387130 | 231 | 76 | + |
| *wMau* | 78.08% ISCca8_aa1 | IS6 | 390516 | 390286 | 231 | 76 | - |
| *wMau* | 65.51% ISSm4_aa2 | ISL3 | 403419 | 402847 | 573 | 190 | - |
| *wMau* | 92.85% ISWpi14_aa3 | IS110 | 403656 | 404675 | 1020 | 339 | + |
| *wMau* | 73.17% ISAcma25_aa2 | IS630 | 419986 | 420237 | 252 | 83 | + |
| *wMau* | 72.94% ISCARN82_aa3 | IS5 ssgr IS1031 | 471622 | 472128 | 507 | 168 | + |
| *wMau* | 58.35% ISGme8_aa1 | IS110 | 509796 | 508831 | 966 | 321 | - |
| *wMau* | 93.15% ISWpi14_aa3 | IS110 | 532883 | 531864 | 1020 | 339 | - |
| *wMau* | 93.15% ISWpi14_aa3 | IS110 | 738255 | 739274 | 1020 | 339 | + |
| *wMau* | 97.46% ISWpi2_aa1 | IS481 | 741077 | 742096 | 1020 | 339 | + |
| *wMau* | 97.56% ISWen1_aa1 | IS4 ssgr IS231 | 815617 | 815210 | 408 | 135 | - |
| *wMau* | 97.71% ISWen1_aa1 | IS4 ssgr IS231 | 816478 | 815945 | 534 | 177 | - |
| *wMau* | 58.35% ISGme8_aa1 | IS110 | 824962 | 823997 | 966 | 321 | - |
| *wMau* | 93.15% ISWpi14_aa3 | IS110 | 826455 | 827552 | 1098 | 365 | + |
| *wMau* | 51.28% ISAba13_aa1 | IS5 ssgr IS903 | 855596 | 855853 | 258 | 85 | + |
| *wMau* | 56.57% ISAcma6_aa1 | IS5 ssgr IS903 | 856197 | 856511 | 315 | 104 | + |
| *wMau* | 93.15% ISWpi14_aa3 | IS110 | 914974 | 913877 | 1098 | 365 | - |
| *wMau* | 58.35% ISGme8_aa1 | IS110 | 928666 | 927701 | 966 | 321 | - |
| *wMau* | 93.15% ISWpi14_aa3 | IS110 | 963290 | 964309 | 1020 | 339 | + |
| *wMau* | 93.15% ISWpi14_aa3 | IS110 | 995344 | 994223 | 1122 | 373 | - |
| *wMau* | 93.15% ISWpi14_aa3 | IS110 | 1060027 | 1058930 | 1098 | 365 | - |
| *wMau* | 93.15% ISWpi14_aa3 | IS110 | 1061951 | 1063048 | 1098 | 365 | + |
| *wMau* | 50.35% IS903_aa1 | IS5 ssgr IS903 | 1142909 | 1143385 | 477 | 158 | + |
| *wMau* | 50.51% ISHph1_aa1 | IS5 ssgr IS903 | 1143493 | 1143849 | 357 | 118 | + |
| *wMau* | 93.15% ISWpi14_aa3 | IS110 | 1198556 | 1199653 | 1098 | 365 | + |
| *wMau* | 93.15% ISWpi14_aa3 | IS110 | 1238303 | 1237206 | 1098 | 365 | - |
| *wAlbB* | 95.83% ISWpi2_aa1 | IS481 | 102 | 608 | 507 | 168 | + |
| *wAlbB* | 96.61% ISWpi2_aa1 | IS481 | 1240 | 635 | 606 | 201 | - |
| *wAlbB* | 84.15% ISWpi16_aa1 | IS982 | 8038 | 7166 | 873 | 290 | - |
| *wAlbB* | 84.50% ISWpi16_aa1 | IS982 | 2239 | 3111 | 873 | 290 | + |
| *wAlbB* | 71.94% ISWen3_aa1 | IS66 ssgr ISBst12 | 45659 | 45222 | 438 | 145 | - |
| *wAlbB* | 84.15% ISWpi16_aa1 | IS982 | 47230 | 46358 | 873 | 290 | - |
| *wAlbB* | 69.95% ISWen3_aa1 | IS66 ssgr ISBst12 | 3970 | 5289 | 1320 | 439 | + |
| *wAlbB* | 96.74% ISWpi2_aa1 | IS481 | 20101 | 19013 | 1089 | 362 | - |
| *wAlbB* | 97.08% ISWpi2_aa1 | IS481 | 21174 | 20167 | 1008 | 335 | - |
| *wAlbB* | 84.50% ISWpi16_aa1 | IS982 | 22926 | 23798 | 873 | 290 | + |
| *wAlbB* | 84.15% ISWpi16_aa1 | IS982 | 25892 | 26764 | 873 | 290 | + |
| *wAlbB* | 96.74% ISWpi2_aa1 | IS481 | 36020 | 34932 | 1089 | 362 | - |
| *wAlbB* | 84.50% ISWpi16_aa1 | IS982 | 47867 | 46995 | 873 | 290 | - |
| *wAlbB* | 96.74% ISWpi2_aa1 | IS481 | 54059 | 52971 | 1089 | 362 | - |
| *wAlbB* | 89.18% ISWpi16_aa1 | IS982 | 1356 | 1820 | 465 | 154 | + |
| *wAlbB* | 84.50% ISWpi16_aa1 | IS982 | 9103 | 8231 | 873 | 290 | - |
| *wAlbB* | 96.74% ISWpi2_aa1 | IS481 | 10402 | 11490 | 1089 | 362 | + |
| *wAlbB* | 96.74% ISWpi2_aa1 | IS481 | 13168 | 12080 | 1089 | 362 | - |
| *wAlbB* | 84.15% ISWpi16_aa1 | IS982 | 45244 | 46116 | 873 | 290 | + |
| *wAlbB* | 74.23% ISHor1_aa2 | IS3 ssgr IS3 | 47641 | 47132 | 510 | 169 | - |
| *wAlbB* | 84.15% ISWpi16_aa1 | IS982 | 61955 | 61083 | 873 | 290 | - |
| *wAlbB* | 84.50% ISWpi16_aa1 | IS982 | 111368 | 112240 | 873 | 290 | + |
| *wAlbB* | 69.73% ISWen3_aa1 | IS66 ssgr ISBst12 | 113751 | 115070 | 1320 | 439 | + |
| *wAlbB* | 96.74% ISWpi2_aa1 | IS481 | 115250 | 116338 | 1089 | 362 | + |
| *wAlbB* | 84.15% ISWpi16_aa1 | IS982 | 119721 | 118849 | 873 | 290 | - |
| *wAlbB* | 84.50% ISWpi16_aa1 | IS982 | 123084 | 123956 | 873 | 290 | + |
| *wAlbB* | 69.23% ISCce4_aa2 | IS3 ssgr IS3 | 135907 | 135308 | 600 | 199 | - |
| *wAlbB* | 68.04% ISHar2_aa1 | IS3 ssgr IS3 | 136461 | 136162 | 300 | 99 | - |
| *wAlbB* | 69.73% ISWen3_aa1 | IS66 ssgr ISBst12 | 140669 | 139350 | 1320 | 439 | - |
| *wAlbB* | 96.74% ISWpi2_aa1 | IS481 | 147525 | 148613 | 1089 | 362 | + |
| *wAlbB* | 84.08% ISWpi16_aa1 | IS982 | 149369 | 148629 | 741 | 246 | - |
| *wAlbB* | 84.15% ISWpi16_aa1 | IS982 | 151185 | 152057 | 873 | 290 | + |
| *wAlbB* | 84.15% ISWpi16_aa1 | IS982 | 162787 | 163659 | 873 | 290 | + |
| *wAlbB* | 96.74% ISWpi2_aa1 | IS481 | 169880 | 170968 | 1089 | 362 | + |
| *wAlbB* | 84.15% ISWpi16_aa1 | IS982 | 187585 | 188457 | 873 | 290 | + |
| *wAlbB* | 84.15% ISWpi16_aa1 | IS982 | 190239 | 191111 | 873 | 290 | + |
| *wAlbB* | 69.83% ISWen3_aa1 | IS66 ssgr ISBst12 | 192287 | 191202 | 1086 | 361 | - |
| *wAlbB* | 84.50% ISWpi16_aa1 | IS982 | 193123 | 193995 | 873 | 290 | + |
| *wAlbB* | 86.17% ISWpi16_aa1 | IS982 | 212214 | 211543 | 672 | 223 | - |
| *wAlbB* | 84.15% ISWpi16_aa1 | IS982 | 213113 | 212241 | 873 | 290 | - |
| *wAlbB* | 82.69% ISWpi16_aa1 | IS982 | 561 | 46 | 516 | 171 | - |
| *wAlbB* | 80.95% ISWpi16_aa1 | IS982 | 730 | 506 | 225 | 74 | - |
| *wAlbB* | 84.15% ISWpi16_aa1 | IS982 | 12475 | 11603 | 873 | 290 | - |
| *wAlbB* | 96.74% ISWpi2_aa1 | IS481 | 27369 | 28457 | 1089 | 362 | + |
| *wAlbB* | 97.20% ISWpi2_aa1 | IS481 | 46794 | 47837 | 1044 | 347 | + |
| *wAlbB* | 84.15% ISWpi16_aa1 | IS982 | 54555 | 53683 | 873 | 290 | - |
| *wAlbB* | 84.15% ISWpi16_aa1 | IS982 | 1818 | 946 | 873 | 290 | - |
| *wAlbB* | 83.70% ISWpi16_aa1 | IS982 | 8026 | 8718 | 693 | 230 | + |
| *wAlbB* | 96.74% ISWpi2_aa1 | IS481 | 9822 | 8734 | 1089 | 362 | - |
| *wAlbB* | 68.04% ISHar2_aa1 | IS3 ssgr IS3 | 13747 | 14046 | 300 | 99 | + |
| *wAlbB* | 68.32% ISHor1_aa2 | IS3 ssgr IS3 | 14043 | 14900 | 858 | 285 | + |
| *wAlbB* | 68.32% ISHor1_aa2 | IS3 ssgr IS3 | 17205 | 16348 | 858 | 285 | - |
| *wAlbB* | 68.04% ISHar2_aa1 | IS3 ssgr IS3 | 17501 | 17202 | 300 | 99 | - |
| *wAlbB* | 96.74% ISWpi2_aa1 | IS481 | 27525 | 26437 | 1089 | 362 | - |
| *wAlbB* | 96.44% ISWpi2_aa1 | IS481 | 37506 | 38594 | 1089 | 362 | + |
| *wAlbB* | 84.32% ISWpi16_aa1 | IS982 | 55795 | 54983 | 813 | 270 | - |
| *wAlbB* | 77.22% ISWpi16_aa1 | IS982 | 76959 | 77297 | 339 | 112 | + |
| *wAlbB* | 84.32% ISWpi16_aa1 | IS982 | 78146 | 77334 | 813 | 270 | - |
| *wAlbB* | 88.06% ISWpi16_aa1 | IS982 | 78263 | 78811 | 549 | 182 | + |
| *wAlbB* | 86.81% ISFw3_aa1 | IS481 | 82622 | 82969 | 348 | 115 | + |
| *wAlbB* | 96.53% ISWpi2_aa1 | IS481 | 83766 | 83068 | 699 | 232 | - |
| *wAlbB* | 84.15% ISWpi16_aa1 | IS982 | 537 | 1409 | 873 | 290 | + |
| *wAlbB* | 77.14% ISWpi16_aa1 | IS982 | 1796 | 2113 | 318 | 105 | + |
| *wAlbB* | 88.63% ISWpi16_aa1 | IS982 | 2120 | 2668 | 549 | 182 | + |
| *wAlbB* | 96.74% ISWpi2_aa1 | IS481 | 15780 | 16868 | 1089 | 362 | + |
| *wAlbB* | 97.20% ISWpi2_aa1 | IS481 | 3577 | 4620 | 1044 | 347 | + |
| *wAlbB* | 96.74% ISWpi2_aa1 | IS481 | 26193 | 27281 | 1089 | 362 | + |
| *wAlbB* | 84.15% ISWpi16_aa1 | IS982 | 31711 | 30839 | 873 | 290 | - |
| *wAlbB* | 84.50% ISWpi16_aa1 | IS982 | 37246 | 36374 | 873 | 290 | - |
| *wAlbB* | 96.38% ISWpi2_aa1 | IS481 | 2919 | 2668 | 252 | 83 | - |
| *wAlbB* | 96.74% ISWpi2_aa1 | IS481 | 4051 | 2963 | 1089 | 362 | - |
| *wAlbB* | 68.04% ISHar2_aa1 | IS3 ssgr IS3 | 9034 | 9333 | 300 | 99 | + |
| *wAlbB* | 68.32% ISHor1_aa2 | IS3 ssgr IS3 | 9330 | 10187 | 858 | 285 | + |
| *wAlbB* | 84.15% ISWpi16_aa1 | IS982 | 14111 | 14983 | 873 | 290 | + |
| *wAlbB* | 96.36% ISWpi2_aa1 | IS481 | 15808 | 15299 | 510 | 169 | - |
| *wAlbB* | 97.95% ISWpi2_aa1 | IS481 | 15870 | 16250 | 381 | 126 | + |
| *wAlbB* | 84.15% ISWpi16_aa1 | IS982 | 1314 | 2186 | 873 | 290 | + |
| *wAlbB* | 84.50% ISWpi16_aa1 | IS982 | 6359 | 7231 | 873 | 290 | + |
| *wAlbB* | 96.53% ISWpi2_aa1 | IS481 | 7352 | 8050 | 699 | 232 | + |
| *wAlbB* | 69.55% ISWen3_aa1 | IS66 ssgr ISBst12 | 8752 | 9957 | 1206 | 401 | + |
| *wAlbB* | 96.44% ISWpi2_aa1 | IS481 | 11056 | 9968 | 1089 | 362 | - |
| *wAlbB* | 96.74% ISWpi2_aa1 | IS481 | 17961 | 16873 | 1089 | 362 | - |
| *wAlbB* | 96.74% ISWpi2_aa1 | IS481 | 24089 | 25177 | 1089 | 362 | + |
| *wAlbB* | 69.95% ISWen3_aa1 | IS66 ssgr ISBst12 | 29119 | 30438 | 1320 | 439 | + |
| *wAlbB* | 96.74% ISWpi2_aa1 | IS481 | 33331 | 32243 | 1089 | 362 | - |
| *wAlbB* | 74.17% ISWen3_aa1 | IS66 ssgr ISBst12 | 35511 | 34927 | 585 | 194 | - |
| *wAlbB* | 84.15% ISWpi16_aa1 | IS982 | 36398 | 35526 | 873 | 290 | - |
| *wAlbB* | 96.96% ISWpi2_aa1 | IS481 | 65 | 763 | 699 | 232 | + |
| *wAlbB* | 95.91% ISWpi2_aa1 | IS481 | 1297 | 779 | 519 | 172 | - |
| *wAlbB* | 69.15% ISWen3_aa1 | IS66 ssgr ISBst12 | 2540 | 1347 | 1194 | 397 | - |
| *wAlbB* | 69.95% ISWen3_aa1 | IS66 ssgr ISBst12 | 22086 | 23405 | 1320 | 439 | + |
| *wAlbB* | 84.15% ISWpi16_aa1 | IS982 | 24624 | 23752 | 873 | 290 | - |
| *wAlbB* | 84.15% ISWpi16_aa1 | IS982 | 30842 | 29970 | 873 | 290 | - |
| *wAlbB* | 96.74% ISWpi2_aa1 | IS481 | 35610 | 34522 | 1089 | 362 | - |
| *wAlbB* | 84.50% ISWpi16_aa1 | IS982 | 37726 | 36854 | 873 | 290 | - |
| *wAlbB* | 83.77% ISWpi16_aa1 | IS982 | 44662 | 45354 | 693 | 230 | + |
| *wAlbB* | 96.74% ISWpi2_aa1 | IS481 | 45420 | 46508 | 1089 | 362 | + |
| *wAlbB* | 96.38% ISWpi2_aa1 | IS481 | 53560 | 53309 | 252 | 83 | - |
| *wAlbB* | 96.74% ISWpi2_aa1 | IS481 | 53720 | 54808 | 1089 | 362 | + |
| *wAlbB* | 96.74% ISWpi2_aa1 | IS481 | 59697 | 58609 | 1089 | 362 | - |
| *wAlbB* | 96.61% ISWpi2_aa1 | IS481 | 60374 | 59763 | 612 | 203 | - |
| *wAlbB* | 96.74% ISWpi2_aa1 | IS481 | 61455 | 62543 | 1089 | 362 | + |
| *wAlbB* | 96.74% ISWpi2_aa1 | IS481 | 69148 | 68060 | 1089 | 362 | - |
| *wAlbB* | 76.47% ISAcma25_aa2 | IS630 | 69195 | 69428 | 234 | 77 | + |
| *wAlbB* | 79.45% ISWpi16_aa1 | IS982 | 79940 | 80380 | 441 | 146 | + |
| *wAlbB* | 89.23% ISWpi16_aa1 | IS982 | 80402 | 80812 | 411 | 136 | + |
| *wAlbB* | 81.25% ISWpi16_aa1 | IS982 | 91882 | 92145 | 264 | 87 | + |
| *wAlbB* | 84.50% ISWpi16_aa1 | IS982 | 1242 | 2114 | 873 | 290 | + |
| *wAlbB* | 96.74% ISWpi2_aa1 | IS481 | 6755 | 5667 | 1089 | 362 | - |
| *wAlbB* | 84.50% ISWpi16_aa1 | IS982 | 18574 | 17702 | 873 | 290 | - |
| *wAlbB* | 96.74% ISWpi2_aa1 | IS481 | 1908 | 2996 | 1089 | 362 | + |
| *wAlbB* | 69.26% ISWen3_aa1 | IS66 ssgr ISBst12 | 3877 | 3155 | 723 | 240 | - |
| *wAlbB* | 84.50% ISWpi16_aa1 | IS982 | 17220 | 18092 | 873 | 290 | + |
| *wAlbB* | 84.50% ISWpi16_aa1 | IS982 | 21012 | 20140 | 873 | 290 | - |
| *wAlbB* | 88.63% ISWpi16_aa1 | IS982 | 46031 | 45483 | 549 | 182 | - |
| *wAlbB* | 77.14% ISWpi16_aa1 | IS982 | 46355 | 46038 | 318 | 105 | - |
| *wAlbB* | 96.74% ISWpi2_aa1 | IS481 | 62191 | 61103 | 1089 | 362 | - |
| *wAlbB* | 96.89% ISWpi2_aa1 | IS481 | 63111 | 62257 | 855 | 284 | - |
| *wAlbB* | 84.15% ISWpi16_aa1 | IS982 | 65026 | 65898 | 873 | 290 | + |
| *wAlbB* | 96.44% ISWpi2_aa1 | IS481 | 69853 | 70941 | 1089 | 362 | + |
| *wAlbB* | 68.04% ISHar2_aa1 | IS3 ssgr IS3 | 74797 | 75096 | 300 | 99 | + |
| *wAlbB* | 96.74% ISWpi2_aa1 | IS481 | 75332 | 76420 | 1089 | 362 | + |
| *wAlbB* | 84.32% ISWpi16_aa1 | IS982 | 78310 | 79122 | 813 | 270 | + |
| *wAlbB* | 68.70% ISHor1_aa2 | IS3 ssgr IS3 | 79174 | 80040 | 867 | 288 | + |
| *wAlbB* | 84.15% ISWpi16_aa1 | IS982 | 1551 | 2423 | 873 | 290 | + |
| *wAlbB* | 84.15% ISWpi16_aa1 | IS982 | 6192 | 5320 | 873 | 290 | - |
| *wAlbB* | 84.50% ISWpi16_aa1 | IS982 | 1168 | 2040 | 873 | 290 | + |
| *wAlbB* | 84.50% ISWpi16_aa1 | IS982 | 7573 | 6701 | 873 | 290 | - |
| *wAlbB* | 84.15% ISWpi16_aa1 | IS982 | 14022 | 14894 | 873 | 290 | + |
| *wAlbB* | 88.63% ISWpi16_aa1 | IS982 | 24413 | 23865 | 549 | 182 | - |
| *wAlbB* | 84.15% ISWpi16_aa1 | IS982 | 2251 | 3123 | 873 | 290 | + |
| *wAlbB* | 84.15% ISWpi16_aa1 | IS982 | 14960 | 15832 | 873 | 290 | + |
| *wAlbB* | 84.50% ISWpi16_aa1 | IS982 | 17034 | 17906 | 873 | 290 | + |
| *wAlbB* | 97.20% ISWpi2_aa1 | IS481 | 34709 | 33666 | 1044 | 347 | - |
| *wAlbB* | 96.74% ISWpi2_aa1 | IS481 | 42560 | 43648 | 1089 | 362 | + |
| *wAlbB* | 84.15% ISWpi16_aa1 | IS982 | 54152 | 53280 | 873 | 290 | - |
| *wAlbB* | 96.74% ISWpi2_aa1 | IS481 | 64751 | 63663 | 1089 | 362 | - |
| *wAlbB* | 84.50% ISWpi16_aa1 | IS982 | 67919 | 67047 | 873 | 290 | - |
| *wAlbB* | 84.15% ISWpi16_aa1 | IS982 | 69106 | 69978 | 873 | 290 | + |
| *wAlbB* | 87.01% ISWpi14_aa1 | IS110 | 74894 | 74487 | 408 | 135 | - |
| *wAlbB* | 96.61% ISWpi2_aa1 | IS481 | 74956 | 75567 | 612 | 203 | + |
| *wAlbB* | 96.74% ISWpi2_aa1 | IS481 | 76682 | 75594 | 1089 | 362 | - |
| *wAlbB* | 88.46% ISWpi15_aa1 | IS256 | 77340 | 77113 | 228 | 75 | - |
| *wAlbB* | 86.44% ISWpi15_aa1 | IS256 | 77509 | 77330 | 180 | 59 | - |
| *wAlbB* | 97.20% ISWpi2_aa1 | IS481 | 84360 | 83317 | 1044 | 347 | - |
| *wAlbB* | 84.15% ISWpi16_aa1 | IS982 | 127008 | 127880 | 873 | 290 | + |
| *wAlbB* | 98.90% ISWosp7_aa1 | IS4 ssgr IS231 | 128288 | 128001 | 288 | 95 | - |
| *wAlbB* | 69.23% ISCce4_aa2 | IS3 ssgr IS3 | 145139 | 144540 | 600 | 199 | - |
| *wAlbB* | 68.04% ISHar2_aa1 | IS3 ssgr IS3 | 145693 | 145394 | 300 | 99 | - |
| *wAlbB* | 84.50% ISWpi16_aa1 | IS982 | 146595 | 145723 | 873 | 290 | - |
| *wAlbB* | 84.15% ISWpi16_aa1 | IS982 | 31140 | 32012 | 873 | 290 | + |
| *wAlbB* | 84.50% ISWpi16_aa1 | IS982 | 39125 | 39997 | 873 | 290 | + |
| *wAlbB* | 69.95% ISWen3_aa1 | IS66 ssgr ISBst12 | 54019 | 52700 | 1320 | 439 | - |
| *wAlbB* | 84.50% ISWpi16_aa1 | IS982 | 65404 | 64532 | 873 | 290 | - |
| *wAlbB* | 96.74% ISWpi2_aa1 | IS481 | 67595 | 68683 | 1089 | 362 | + |
| *wAlbB* | 96.74% ISWpi2_aa1 | IS481 | 70272 | 69184 | 1089 | 362 | - |
| *wAlbB* | 84.15% ISWpi16_aa1 | IS982 | 80305 | 81177 | 873 | 290 | + |
| *wAlbB* | 76.78% ISXo3_aa1 | IS5 ssgr IS1031 | 91740 | 91534 | 207 | 68 | - |
| *wAlbB* | 84.15% ISWpi16_aa1 | IS982 | 95090 | 95962 | 873 | 290 | + |
| *wAlbB* | 68.04% ISHar2_aa1 | IS3 ssgr IS3 | 120135 | 120434 | 300 | 99 | + |
| *wAlbB* | 84.15% ISWpi16_aa1 | IS982 | 120541 | 121413 | 873 | 290 | + |
| *wAlbB* | 96.96% ISWpi2_aa1 | IS481 | 124358 | 123660 | 699 | 232 | - |
| *wAlbB* | 84.15% ISWpi16_aa1 | IS982 | 2454 | 3326 | 873 | 290 | + |
| *wAlbB* | 84.50% ISWpi16_aa1 | IS982 | 16327 | 15455 | 873 | 290 | - |
| *wAlbB* | 84.50% ISWpi16_aa1 | IS982 | 17394 | 16522 | 873 | 290 | - |
| *wAlbB* | 69.73% ISWen3_aa1 | IS66 ssgr ISBst12 | 19029 | 17710 | 1320 | 439 | - |
| *wAlbB* | 86.15% ISWpi16_aa1 | IS982 | 21111 | 20896 | 216 | 71 | - |
| *wAlbB* | 84.15% ISWpi16_aa1 | IS982 | 22080 | 21208 | 873 | 290 | - |
| *wAlbB* | 87.93% ISWpi16_aa1 | IS982 | 38312 | 38112 | 201 | 66 | - |
| *wAlbB* | 96.74% ISWpi2_aa1 | IS481 | 38391 | 39479 | 1089 | 362 | + |
| *wAlbB* | 84.50% ISWpi16_aa1 | IS982 | 41584 | 42456 | 873 | 290 | + |
| *wAlbB* | 84.50% ISWpi16_aa1 | IS982 | 55200 | 56072 | 873 | 290 | + |
| *wAlbB* | 84.15% ISWpi16_aa1 | IS982 | 62068 | 61196 | 873 | 290 | - |
| *wAlbB* | 84.50% ISWpi16_aa1 | IS982 | 79809 | 78937 | 873 | 290 | - |
| *wAlbB* | 69.73% ISWen3_aa1 | IS66 ssgr ISBst12 | 83508 | 82189 | 1320 | 439 | - |
| *wAlbB* | 97.20% ISWpi2_aa1 | IS481 | 98902 | 99945 | 1044 | 347 | + |
| *wAlbB* | 96.74% ISWpi2_aa1 | IS481 | 104281 | 105369 | 1089 | 362 | + |
| *wAlbB* | 70.62% ISWen3_aa1 | IS66 ssgr ISBst12 | 105455 | 106735 | 1281 | 426 | + |
| *wAlbB* | 96.74% ISWpi2_aa1 | IS481 | 106801 | 107889 | 1089 | 362 | + |
| *wAlbB* | 97.20% ISWpi2_aa1 | IS481 | 124090 | 125133 | 1044 | 347 | + |
| *wAlbB* | 96.74% ISWpi2_aa1 | IS481 | 135619 | 134531 | 1089 | 362 | - |
| *wAlbB* | 96.74% ISWpi2_aa1 | IS481 | 141490 | 142578 | 1089 | 362 | + |
| *wAlbB* | 84.50% ISWpi16_aa1 | IS982 | 144250 | 143378 | 873 | 290 | - |
| *wAlbB* | 96.75% ISWpi2_aa1 | IS481 | 896 | 357 | 540 | 179 | - |
| *wAlbB* | 96.46% ISWpi2_aa1 | IS481 | 943 | 1566 | 624 | 207 | + |
| *wAlbB* | 96.74% ISWpi2_aa1 | IS481 | 8646 | 9734 | 1089 | 362 | + |
| *wAlbB* | 68.32% ISHor1_aa2 | IS3 ssgr IS3 | 14520 | 13663 | 858 | 285 | - |
| *wAlbB* | 68.04% ISHar2_aa1 | IS3 ssgr IS3 | 14816 | 14517 | 300 | 99 | - |
| *wAlbB* | 97.33% ISWpi2_aa1 | IS481 | 16321 | 16848 | 528 | 175 | + |
| *wAlbB* | 96.74% ISWpi2_aa1 | IS481 | 16914 | 18002 | 1089 | 362 | + |
| *wAlbB* | 96.74% ISWpi2_aa1 | IS481 | 29093 | 30181 | 1089 | 362 | + |
| *wAlbB* | 96.96% ISWpi2_aa1 | IS481 | 36509 | 35811 | 699 | 232 | - |
| *wAlbB* | 97.93% ISWpi2_aa1 | IS481 | 36898 | 36530 | 369 | 122 | - |
| *wAlbB* | 96.74% ISWpi2_aa1 | IS481 | 45268 | 44180 | 1089 | 362 | - |
| *wAlbB* | 96.74% ISWpi2_aa1 | IS481 | 46227 | 47315 | 1089 | 362 | + |
| *wAlbB* | 88.37% ISWpi16_aa1 | IS982 | 66041 | 65604 | 438 | 145 | - |
| *wAlbB* | 84.50% ISWpi16_aa1 | IS982 | 72781 | 73653 | 873 | 290 | + |
| *wAlbB* | 84.15% ISWpi16_aa1 | IS982 | 90082 | 89210 | 873 | 290 | - |
| *wAlbB* | 69.26% ISWen3_aa1 | IS66 ssgr ISBst12 | 92763 | 92041 | 723 | 240 | - |
| *wAlbB* | 68.32% ISHor1_aa2 | IS3 ssgr IS3 | 109191 | 108334 | 858 | 285 | - |
| *wAlbB* | 68.04% ISHar2_aa1 | IS3 ssgr IS3 | 109487 | 109188 | 300 | 99 | - |
| *wAlbB* | 84.50% ISWpi16_aa1 | IS982 | 110168 | 111040 | 873 | 290 | + |
| *wAlbB* | 96.74% ISWpi2_aa1 | IS481 | 119471 | 118383 | 1089 | 362 | - |
| *wAlbB* | 84.15% ISWpi16_aa1 | IS982 | 133922 | 134794 | 873 | 290 | + |
| *wAlbB* | 82.45% ISWpi16_aa1 | IS982 | 142058 | 141855 | 204 | 67 | - |
| *wAlbB* | 82.11% ISWpi16_aa1 | IS982 | 142702 | 142055 | 648 | 215 | - |
| *wAlbB* | 84.50% ISWpi16_aa1 | IS982 | 163756 | 164628 | 873 | 290 | + |
| *wAlbB* | 90.42% ISWpi2_aa1 | IS481 | 164955 | 164572 | 384 | 127 | - |
| *wAlbB* | 71.94% ISWen3_aa1 | IS66 ssgr ISBst12 | 169832 | 169395 | 438 | 145 | - |
| *wAlbB* | 84.50% ISWpi16_aa1 | IS982 | 171938 | 172810 | 873 | 290 | + |
| *wAlbB* | 84.50% ISWpi16_aa1 | IS982 | 186316 | 185444 | 873 | 290 | - |
| *wAlbB* | 96.66% ISWpi2_aa1 | IS481 | 188303 | 189028 | 726 | 241 | + |
| *wAlbB* | 96.74% ISWpi2_aa1 | IS481 | 217313 | 216225 | 1089 | 362 | - |
| *wAlbB* | 83.95% ISWpi16_aa1 | IS982 | 231675 | 230863 | 813 | 270 | - |
| *wAlbB* | 69.18% ISWen3_aa1 | IS66 ssgr ISBst12 | 239541 | 240083 | 543 | 180 | + |
| *wAlbB* | 84.50% ISWpi16_aa1 | IS982 | 240155 | 241027 | 873 | 290 | + |

**Supplementary Table S2:** Confirmation of SNPs between the assembly of the current study and *wBm* reference (ASM838v1;GCF_000008385.1).

| **position (bp)** | **ref wBm** | **produced wBm** | **mutation synonym** | **region ID** | **region name** | **wWb (ASM220423v2)** | **PCR** |
| --- | --- | --- | --- | --- | --- | --- | --- |
| 678,738 | G | T | Pseudo. | Wbm5063 | Drug resistance transporter Bcr/CflA family | T (NJBR02000038) | yes, T |
| 678,739 | G | T | Pseudo. | Wbm5063 | Drug resistance transporter Bcr/CflA family | T (NJBR02000038) | yes, T |
| 678,826 | G | T | Pseudo. | Wbm5063 | Drug resistance transporter Bcr/CflA family | C (NJBR02000038) | yes, T |
| 678,827 | G | T | Pseudo. | Wbm5063 | Drug resistance transporter Bcr/CflA family | T (NJBR02000038) | yes, T |
| 692,659 | C | G | no (P/D) | Wbm0534 | Tryptophanyl-tRNA synthetase | G (NJBR02000038) | yes, G |
| 692,660 | C | A | no (P/D) | Wbm0534 | Tryptophanyl-tRNA synthetase | A (NJBR02000038) | yes, A |
| 695,065 | G | T | no (P/T) | Wbm0535 | 4-hydroxybenzoate polyprenyltransferase | T (NJBR02000038) | yes, T |
| 695,066 | G | A | no (P/T) | Wbm0535 | 4-hydroxybenzoate polyprenyltransferase | A (NJBR02000038) | yes, A |
| 736,445 | - | A | no | Wbm0560 | PQQ-like beta-propeller repeat-containing protein | A (NJBR02000002) | yes, A |
| 832,790 | C | T | no (C/Y) | Wbm0636 | tRNA delta(2)-isopentenylpyrophosphate transferase | T (NJBR02000093) | yes, T |
| 834,108 | G | A | no (G/D) | Wbm0638 | Putative translation factor, SUA5 | A (NJBR02000093) | yes, A |
| 837,797 | G | A | no (A/V) | Wbm0643 | UDP-N-acetylmuramyl pentapeptide phosphotransferase | A (NJBR02000093) | yes, A |
| 838,394 | G | A | no (T/M) | Wbm0644 | DNA segregation ATPase FtsK | A (NJBR02000093) | yes, A |
| 948,208 | G | A | no (G/R) | Wbm0725 | DNA polymerase I, PolA | A (NJBR02000006) | yes, A |
| 948,606 | C | T | yes | Wbm0725 | DNA polymerase I, PolA | T (NJBR02000006) | yes, T |
| 948,889 | G | A | no (E/K) | Wbm0726 | DNA polymerase I, PolA | A (NJBR02000006) | yes, A |
| 949,008 | A | G | yes | Wbm0725 | DNA polymerase I, PolA | G (NJBR02000006) | yes, G |
| 951,478 | C | A | no (H/Q) | Wbm0726 | predicted protein | A (NJBR02000040) | yes, A |

**Supplementary Table S3:** Observed base deletions between the assembly of the current study and *wBm* reference (ASM838v1;GCF_000008385.1).

| **position (bp)** | **ref *wBm*** | **produced *wBm*** | **region ID** | **region name** | ***wWb* (ASM220423v2)** |
| --- | --- | --- | --- | --- | --- |
| 15,432 | C | - | Wbm7001 | tRNA-Ser | C (NJBR02000022) |
| 71,707 | T | - | Wbm0051 | NADPH-dependent glutamate synthase beta chain | T (NJBR02000014) |
| 98,858 | C | - | Wbm0076 | WAS family protein | T (NJBR02000073) |
| 210,662 | G | - |  | URR_36 unclassified repeat region | del (NJBR02000016) |
| 246,135 | G | - | Wbm0192 | Nucleotide-binding protein | G (NJBR02000097) |
| 278,245 | G | - | Wbm0209 | Phosphoenolpyruvate synthase/pyruvate phosphate dikinase | A ( NJBR02000007) |
| 285,148 | G | - | Wbm0215 | Uncharacterized conserved protein | A (NJBR02000099) |
| 323,752 | A | - | Wbm0256 | 5-formyltetrahydrofolate cyclo-ligase | C (NJBR02000024) |
| 331,035 | G | - | Wbm0260 | Dioxygenases related to 2-nitropropane dioxygenase | G (NJBR02000024) |
| 335,824 | G | - | Wbm0263 | Excinuclease ATPase subunit, UvrA | A (NJBR02000024) |
| 391,236 | G | - |  | URR_62 unclassified repeat region | A (NJBR02000053) |
| 428,849 | T | - | Wbm0338 | Ribosomal protein L2 | C (NJBR02000003) |
| 486,611 | T | - | no coding | - | C (NJBR02000009) |
| 580,782 | C | - | Wbm0446 | Dihydroorotase, PyrC | T (NJBR02000090) |
| 855,047 | A | - | Wbm7024 | tRNA-Gly | del (ADHD01000046) |
| 873,127 | C | - | Wbm0668 | Malonyl-CoA decarboxylase | A (NJBR02000062) |
| 960,580 | T | - | Wbm0731 | DnaA paralog | del (NJBR02000040) |
| 974,597 | C | - | Wbm0744 | Predicted protein | T (NJBR02000048) |
| 978,829 | T | - |  | URR_166 unclassified repeat region | T (NJBR02000026) |
| 1,051,801 | T | - | Wbm0792 | Predicted protein | C (NJBR02000047) |
| 1,075,210 | C | - | Wbm0808 | Ribosomal protein S2 | T (NJBR02000027) |

**Supplementary Table S4:** Confirmation of difference between the two *wMau* assemblies of the current study (from population 181 and population 177 flies).

| **position (bp)** | **base wMau p181** | **base wMau p177** | **mutation synonym** | **region** | **similarity with closest *wb*, *wNo*** | **PCR** |
| --- | --- | --- | --- | --- | --- | --- |
| 31,334 | T | - | no | hypothetical protein | T (WP_015588153) | NA |
| 129,541 | C | - | no | ribosome biogenesis GTPase | C (WP_015588106) | NA |
| 337,799 | C | - | no | NADP-dependent malic enzyme | C (WP_015588375) | NA |
| 547,242 | - | T | no | hypothetical protein | T (AGJ99367) | NA |
| 593,774 | A | G | no K (p181) or E (p177) | SpvB and TcdB toxin domain protein | G (WP_015588516) | NA |
| 595,636 | - | A | no | SpvB and TcdB toxin domain protein | A (WP_015588516) | NA |
| 598,821 | A | - | no | hypothetical protein | A (WP_081600826) | NA |
| 601,629 | - | T | no | hypothetical protein | T (AGJ99403) | yes, T |
| 602,827 | - | C | no | hypothetical protein | C (AGJ99403) | NA |
| 602,882 | - | A | no | hypothetical protein | A (AGJ99403) | NA |
| 604,084 | C | - | no | hypothetical protein | C (WP_081600826) | NA |
| 605,246 | A | - | no | hypothetical protein | A (WP_081600826) | NA |
| 700,765 | - | C | no | Ankyrin repeat domain protein | C (WP_015588545) | NA |
| 703,365 | - | A | no | Ankyrin repeat domain protein | A (WP_015587816) | NA |
| 706,037 | G | - | no | hypothetical protein, pseudogene | G (CP003883, locus wNo_10620) | NA |
| 706,890 | C | - | no | hypothetical protein, collagen-like protein | low similarity NA | NA |
| 770,421 | G | - | no | Ankyrin repeat domain protein | G (AGJ98480) | NA |
| 806,954 | G | - | no | Ankyrin repeat domain protein | G (WP_015588599) | yes, G |
| 936,093 | N | C | no | Ribosomal RNA small subunit methyltransferase A | C (AGJ98633) | yes, C |

**Supplementary Table S5:** PCR condition and results testing potential SNPs between assemblies and references. The three first columns describe the SNP at the reference position. Columns 4 to 6 described sequences of the primers for PCR and the temperature of the annealing step. General PCR conditions 1X Q5® Hot Start High-Fidelity Master Mix (New England Biolabs) and 1μM primers; 2 min 98˚C, 30 cycles of 98˚C 20s, 66-70˚C 30s, 68˚C 30s, then 5 min 72˚C. The two last columns describe the size of the sequenced amplicons and the observed base.

| **Ref** | **position (bp)** | **Base ref** | **primers** | | | **amplicon** | |
| --- | --- | --- | --- | --- | --- | --- | --- |
|  |  |  | **Forward sequence** | **Reverse sequence** | **T ˚C** | **size** | **base** |
| *wBm* | 678,738 | G | ATGGCCAGAATGTGAAGGCTCAA | TCTTCCTAACACCATACCGGAGCA | 68 | 601 | T |
| *wBm* | 678,739 | G |  |  |  |  | T |
| *wBm* | 678,826 | G |  |  |  |  | T |
| *wBm* | 678,827 | G |  |  |  |  | T |
| *wBm* | 692,659 | C | CAAGTCATCTGTGTCGTCAAGG | CCAGTTGGTTGGCTTAATCG | 68 | 276 | G |
| *wBm* | 692,660 | C |  |  |  |  | A |
| *wBm* | 695,065 | G | ATCCTACAGCAAGCAGAGCA | TGGCCACAATTGTTTCTAGGGT | 68 | 260 | T |
| *wBm* | 695,066 | G |  |  |  |  | A |
| *wBm* | 736,445 | - | TGAGAGCTCCTGCAAGAGGAA | ACCTTTAGTGGCTTGGACCAT | 68 | 333 | A |
| *wBm* | 832,790 | C | CGAAAATTGACTTGCCAGTTGC | ACAGGAATCACAGCTTCAGG | 68 | 428 | T |
| *wBm* | 834,108 | G | CACTTTTCTCCCGGACCAATTACC | ACCGCAGATTAACCAAGATTGAAC |  | 369 | A |
| *wBm* | 837,797 | G | GTCGCTCCTATAAACGTGATGC | TCCTCTGCGGAACTAAAAGGTGA | 68 | 834 | A |
| *wBm* | 838,394 | G |  |  |  |  | A |
| *wBm* | 948,208 | G | ATCGATGGTTACGGCTTTCT | GGCAATGAATTGCGACTTTTCC | 66 | 836 | A |
| *wBm* | 948,606 | C |  |  |  |  | T |
| *wBm* | 948,889 | G |  |  |  |  | A |
| *wBm* | 949,008 | A |  |  |  |  | G |
| *wBm* | 951,478 | C | TCTGGAATCCTCAAAGCCATCTAC | ACTGACACACTCAGCTACGACT | 68 | 383 | A |
| *wMau* | 593,774 | A or G | AGATGCACTGGACAAAGCG | CACTTCCTGCTCAAGATCTG | 68 | 605 | A |
| *wMau* | 601,629 | T or - | TTTGAGGATTTTGCCCAGCTTGTG | ATTGCTTAGCTCGCCCAGTACTTC | 70 | 1,074 | T |
| *wMau* | 806,954 | G or - | TTGGGCCAGTGGAAGGAGATATTG | GCATGACGTAAAGGAGTCCAAC | 68 | 1,627 | G |
| *wMau* | 936,093 | N or C | GGGCAATAGCTATTGCAAAGCCTG | CTACCAATTGAGCTAAGCCGGCAT | 70 | 1,090 | C |
